# Transoral robotics in otolaryngology: a new frontier to be conquered

**DOI:** 10.1016/j.bjorl.2022.08.001

**Published:** 2022-08-19

**Authors:** Adriano Santana Fonseca

**Affiliations:** a Nicholson Center, Orlando, United States; b Oncoclinicas, Santa Casa Bahia, Salvador, BA, Brazil

The term “robotics” was initially written by Isaac Asimov in his fictional paper “Robot Stories”.^1^ Robotic surgery consists in using an electromechanical device, handled by a trained surgeon to control computerized arms.^2^ Robotic surgery provides access to surgical sites with limited visualization, empowered by a three-dimensional view and instruments that allow to work in areas with limited movements.

Approved by the FDA since 2009, the use of the robot surgery dated back in 1993. However, most of scientific evidence was published in the last 3 years. Literature review from 2021, identified 154 clinical trials, three of them in otolaryngology and 43 randomized.^3^

Unlike other surgical specialties, otolaryngologists still do not incorporate robotics as a standard surgical procedure. The fact that robots were developed for abdominal surgery, without focusing on otolaryngology, caused the specialty to adapt using those robotic platforms for transoral procedures all over the world. However, the technique resulted in great advantages for patients and surgeons.

In addition to the advantages already mentioned, it was observed over time that transoral robotic surgery (TORS) developed more standardized procedures, facilitating both reproducibility and teaching.

## Clinical application

### Oropharyngeal malignant diseases

Transoral robotic surgery offers advantages over more aggressive procedures for well-selected patients. TORS benefits are greater for patients whom surgical resection can reduce or eliminate the need for adjuvant therapy, open approaches, or primary chemoradiation.^3^ Transoral laser surgery uses the same transoral approach, but is limited by tangential cuts, risks of burning the airway, and limited hemostasis. Furthermore, laser technique turns difficult “en bloc” resection of tumors. In this case, TORS by its aforementioned resources, offers advantages over other surgical techniques.

### Neck metatases of an unknown primary

TORS has also become a valuable tool in the investigation of patients with CUP. Palatine tonsillectomy and robotic lingual mucosectomy allows the identification of the primary carcinoma site in 72%–82% of patients, with a consequent reduction in toxicity of chemoradiation. Literature have shown that TORS can provide a complete tumor removal in 78% of patients.^3^

### Treatment of laryngeal disease

TORS is applied successfully for supraglottic surgeries, both in benign and malignant pathologies. Total robotic laryngectomies were previously described, however, the access to the glottis and sub glottis is limited without the single port technology (SP).

### Obstructive sleep apnea (OSAS) and snoring

The best treatment strategy for sleep apnea is determined by a good patient selection. Continue positive airway pressure (CPAP), despite proved to be effective, has low long-term adherence (4). TORS was initially applied in 2010 as an alternative treatment for OSA, addressing the oro/hypopharyngeal obstruction.^4^ Tongue base reduction (BOT) and supraglottoplasty is the primary focus of TORS for OSA treatment. Resections can include the lingual tonsil and the tongue base, including an epiglottoplasty in selected cases.^5^ The main advantages of TORS, compared to non-robotic surgery, are the three-dimensional, and detailed visualization of this area, allowing a safe dissection and vascular control in each surgical step.^4^

### Skull base and nasopharynx

O'Malley and Weinstein, in 2007, described TORS for skull base surgery. The first clinical application occurred in 2012 for the resection of a recurrent nasopharyngeal carcinoma with a nasal and oral approach combined. Since then, there have been several case reports that describe the use of robotics techniques for pathologies in the skull base. The robotic system has an advantage when compared with an endoscope, allowing both hands-free, independent optics motion, and three-dimensional visualization but still limited by narrow space, size of arms, and limited tools.

## Future

Robotic technologies are evolving continuously, adding haptic feedback and augmented reality with microscopic images. Advances in robotic technologies are certainly moving towards filling the needs of all surgical specialties. Otolaryngology and head and neck surgery are already advancing in robotics for both transoral and cervical remote access. Although the costs were initially high, they are nowadays comparable to other ENT technologies as the robots become popular among other specialties. Robotic system may replace endoscopic surgery platforms and have a major influence on how we perform ENT surgery in a near future, as has happened in the past with the endoscopic surgery. Otolaryngologists assist many patients who can benefit from this technique for procedures addressing both benign and malignant diseases. The increase in number of robot-assisted procedures can offer to otolaryngologists training and the required experience to master the future of transoral robotic surgery.

## Conflicts of interest

The author declares no conflicts of interest.
